# Interface topology for distinguishing stages of sintering

**DOI:** 10.1038/s41598-017-11667-2

**Published:** 2017-09-11

**Authors:** Gaku Okuma, Daiki Kadowaki, Tsuyoshi Hondo, Satoshi Tanaka, Fumihiro Wakai

**Affiliations:** 1 Laboratory for Materials and Structures, Institute of Innovative Research, Tokyo Institute of Technology, R3-23 4259 Nagatsuta, Midori, Yokohama 226-8503 Japan; 2 Department of Materials Science and Technology, Nagaoka University of Technology, 1603-1, Kamitomioka, Nagaoka, Niigata 940-2188 Japan

## Abstract

Sintering is a common process during which nanoparticles and microparticles are bonded, leading to the shrinkage of interstitial pore space. Understanding morphological evolution during sintering is a challenge, because pore structures are elusive and very complex. A topological model of sintering is presented here, providing insight for understanding 3-D microstructures observed by X-ray microtomography. We find that the topological evolution is described by Euler characteristics as a function of relative density. The result is general, and applicable not only to viscous sintering of glasses but also to sintering of crystalline particles. It provides criteria to distinguish the stages of sintering, and the foundations to identify the range of applicability of the methods for determining the thermodynamic driving force of sintering.

## Introduction

Sintering of small particles is common in nature, and provides an engineering process for the production of ceramics, metals, glasses, polymers and composites. A huge number of particles undergoes a change in shape at elevated temperatures by matter transport driven by surface tension, the kinetics of which is controlled by viscous flow for amorphous particles^1–3^ or diffusion mechanisms for crystalline particles^4–6^. Since sintering occurs so as to decrease the total interface energy, surface area per unit volume decreases with time. Concurrently, the total pore volume decreases, thereby, the sintering process is described as densification using the relative density as a state parameter. Coble^5^ illustrated the evolution of particle-pore structure schematically, and identified three stages of sintering. The initial stage is characterized as the formation and growth of contact between neighboring particles, where relative density increases from 0.5 to 0.6. In the intermediate stage, the pore structure evolves into an interconnected channel with cylindrical pores lying primarily along three grain edges. The final stage begins when the pinch-off of interconnected pore channel forms closed pores at the relative density of 0.9. The final stage of sintering of crystalline particles is usually accompanied by coarsening and grain growth^7, 8^. Classical sintering theories, which predict the rate of density change, have been proposed with the assumption of simplified geometrical model for each stage^5, 6^.

Recent advances in X-ray microtomography revealed that the three-dimensional (3D) microstructural evolution during sintering is far more complicated than the simplified model. This limits the applicability of classical models in real situations. The direct measurement of a 3D structure, which is now readily available from X-ray microtomography, provides a basis for the statistical analysis of microstructural characteristics, such as relative density, specific surface area, surface curvature, particle size, neck radius^9, 10^, coordination number^10^, heterogeneous particle displacement^11, 12^, particle rotation^13^, pore orientation^14^, pore coarsening^15, 16^, grain growth^17^, and microstructural anisotropy^18^. The knowledge of microstructure is the first step to understand the realistic property-microstructure relationship during sintering.

Macroscopically, shrinkage is a response of porous body to mechanical stress and a thermodynamic driving force, i.e., sintering stress^19, 20^. The shrinkage rate is inversely proportional to the bulk viscosity. The macroscopic quantities such as sintering stress^21–23^ and bulk viscosity^20, 24^ can be determined rigorously for some idealized microstructures in equilibrium. However, for real porous structures which are nonequilibrium, non-periodic, and nonuniform, it is still a challenge to estimate macroscopic quantities from microstructures observed by X-ray microtomography^25–27^. It is recognized that the most appropriate method for determining sintering stress should be selected depending on sintering stage^27^. However, no theoretical criterion exists for distinguishing stages of sintering by using relative density and specific surface area, which are metric properties and vary monotonically during sintering.

Alternatively, topological properties are required to quantify the complex microstructural changes in three stages of sintering. Rhines, DeHoff, and Aigeltinger^28–30^ made a pioneering attempt to analyze the topological properties (e.g., the connectivity or genus per unit volume, and the number of pores per unit volume) by using a stereological method. But, their analysis has not been used widely in the sintering community due to the difficulty in the analysis of two dimensional cross sectional data. The aim of the present work is to obtain quantitative knowledges on topological properties, which provide insights for distinguishing stages in sintering. The microstructural evolution in sintering is an example of morphogenesis, defined as the ensemble of mechanisms responsible for the formation of patterns and shapes^31^. The observation of interface topology by X-ray microtomography affects our thinking on sintering significantly. While most of sintering studies are concerned with distinguishing matter transport mechanisms, we show that the evolution of interface topology shows remarkable similarity between viscous sintering of glass and diffusional sintering of crystalline particles.

## Results

### Microstructural evolution in sintering

**Figure 1:**
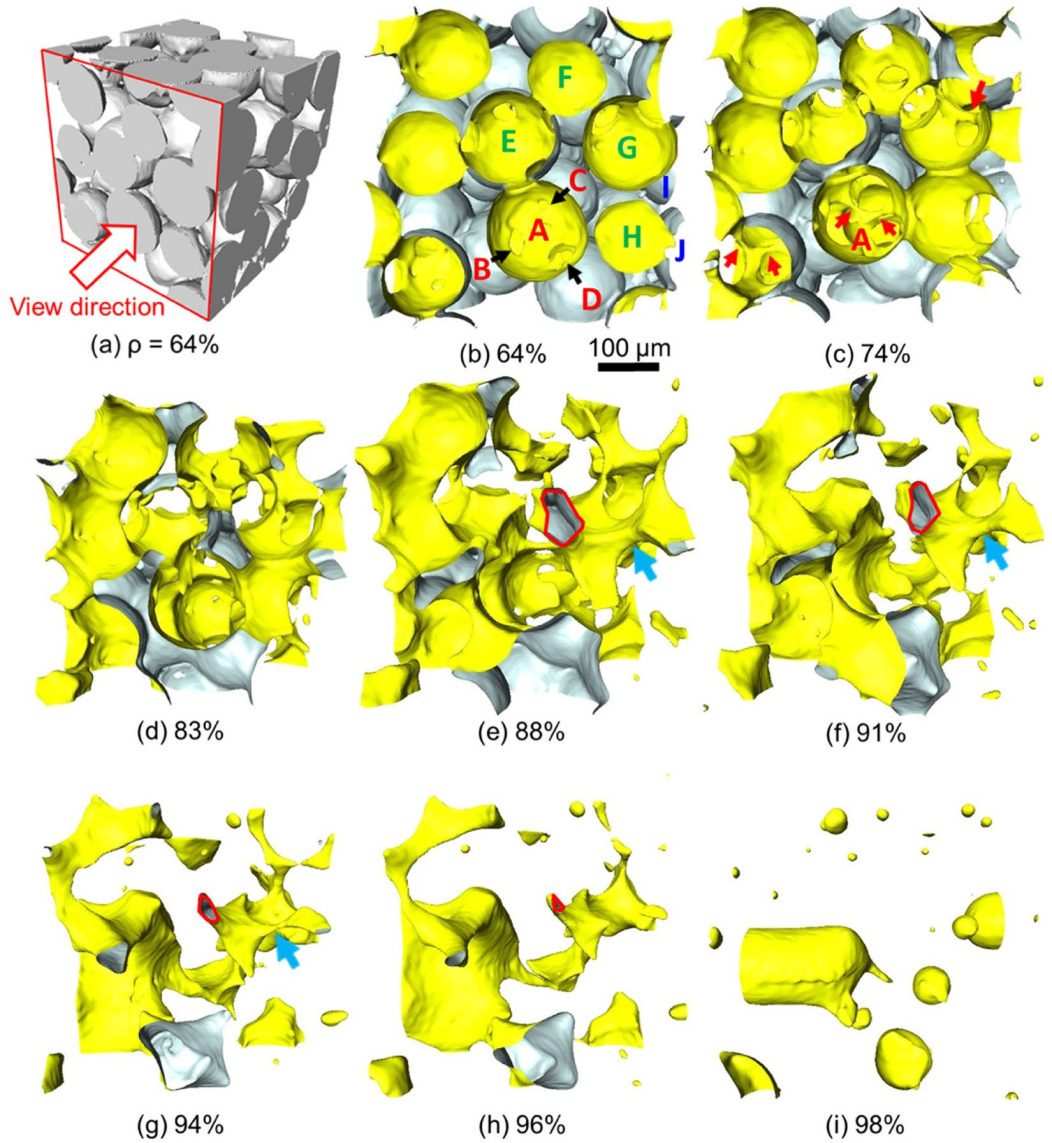
Microstructural evolution in viscous sintering of spherical glass particles. (**a**) solid phase at the initial relative density ρ of 64%, (**b**–**i**) pore space view as seen in the direction of arrow in (**a**). Particle surfaces are shown in white, while pore surfaces are shown in yellow. The size of the reconstructed subvolume is 500 × 500 × 500 µm.

The viscous sintering of spherical glass particles was observed by X-ray microtomography (see Methods). The microstructural evolution is illustrated in Fig. 1 and Supplementary Movies 1 and 1c. In the loose packing of particles (relative density ρ = 64%, Fig. 1a), individual particles have contacts with neighbor particles, but some neighbors are not touching yet. In the pore space view (Fig. 1b), particles, which intersect with the bounding box, are seen as concave surface, where circular apertures indicate contacts with neighbor particles. For example, in sintering of a cluster of four particles (A, B, C, and D), three apertures (arrows) can be seen on the surface of particle A.

**Figure 2:**
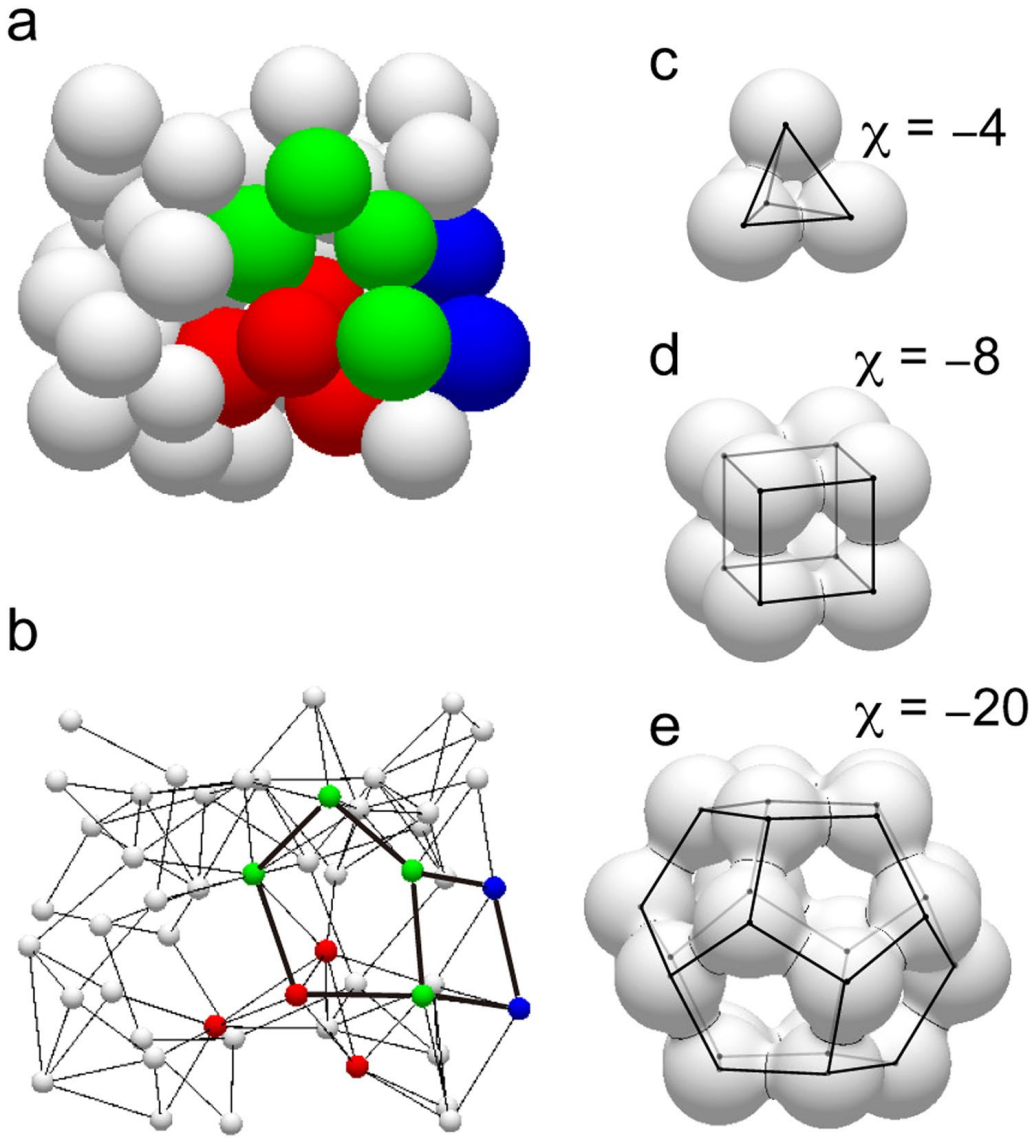
Packing model of spherical particles. (**a**) Sphere packing at ρ = 74%. (**b**) The bond network model consisting of vertices, edges, faces, and cells. A quadrilateral face and a pentagonal face are shown for example. Some polyhedral cells ((**c**) tetrahedron, (**d**) cube, and (**e**) dodecahedron) illustrate that voids inside cells are connected by pore channel (or hole) at the center of each face.

The arrangement of spherical particles reconstructed from a tomography image at ρ = 74% is illustrated in Fig. 2a (see also Supplementary Movie 2a), and the topology of solid phase is schematically shown in Fig. 2b (see also Supplementary Movie 2b). A point (node, vertex) represents each particle. A contact between two particles is represented by an arc (branch, edge) between the representative points. A face is defined by a polygon composed of these vertices and edges. A cell is defined by a 3-D space partitioned by these faces^32^. There is a void in each cell, from which a closed pore may be formed during sintering. The irregular bond network can be characterized by a combination of faces and partial polyhedral cells. Some local structure models depicted in Fig. 2c–e consist of triangular, rectangular, and pentagonal faces, respectively. There is a hole at the center of each face, then, the porous solid phase structure is a continuous network with numerous holes. In the pore space view, this structure is represented as voids inside cells which are connected by pore channels passing through faces.

The cluster of four particles (Fig. 2c) is indeed observed by X-ray microtomography (particles A, B, C, and D in Fig. 1b). As the contact radius (i.e., neck radius) grows with time, the size of a hole in the ring of three particles becomes smaller, and can be seen as a pore channel or a ligament (red arrows in Fig. 1c). The breakup of pore channel among three particles (A, B, and D) has already occurred in Fig. 1c. When two remaining pore channels are pinched off, a single small closed pore is formed at the center of the tetrahedral particle cluster. But, the formed pore disappears quickly. The shrinkage of a tetrahedral pore, which is the characteristics of the “final stage” in the classical sintering model, takes place in very early stage actually.

The cross section of a pore channel is a polygon with rounded corners, where the number of edges is the number of particles surrounding the channel. For example, the pore channel outlined in red (Fig. 1e) is formed by five particles (A, E, F, G, H). The size of the pore channel decreases with densification, and becomes zero at the pinch-off finally. The initial size of a pore channel usually increases with the number of particles surrounding it. The pore channel formed by the ring of three spheres has a small initial size, and can be seen in the early stage (red arrows, Fig. 1c). But, such pore channel along three-grain junction is pinched off quickly. Large pore channels surrounded by many particles are important in the intermediate stage. An example of pore channel formed by the ring of four spheres (G, H, I, J) is seen in Fig. 1e (blue arrow). Such large pore channel remains up to the relative density of 94% before the pinch-off (Fig. 1g). When a pore channel is pinched off, one hole is closed, thereby decreasing the number of holes.

Many tetrahedral cells form small closed pores by the pinch-off of pore channels, and these small tetrahedral pores shrink and disappear quickly. The size of a cell, and its void space, usually increases with the number of particles surrounding it, or pore coordination number^33^. The closed pores larger than the particle size are formed from large voids preexisting in the random packing of particles. Such voids are connected with open pore channels initially, and are separated later through consecutive pinch-offs. Closed pores formed by viscous sintering in air become spherical (Fig. 1i), and shrink by gas diffusion in the glass. The number of spherical pores decreases to zero ultimately in the final stage of sintering.

### Euler characteristic for describing interface topology

The naturally evolving interface changes its topology during sintering. The topological transitions are summarized as follows: (1) Formation of contacts among particles leading to the increase of number of holes *G* in the networks, (2) Pinch-off of pore channels, i.e., the decrease of *G* by the closure of holes, (3) Formation of closed pores, i.e., the increase of number of pores *N*, (4) Disappearance of closed pores, i.e., the decrease of *N*. The term “holes” is used for pore channels between porous cells throughout this paper, then, “hole closure” means the pinch-off of pore channel. The topology of a surface is characterized by its genus *g*; roughly speaking it is the number of holes in the surface. A single sphere has *g* = 0, and a torus (doughnut shape) has *g* = 1. The genus is mathematically related to the Euler characteristic as *χ* = 2 − 2 *g*. For partially sintered particle clusters, tetrahedron (Fig. 2c), cube (Fig. 2d), and dodecahedron (Fig. 2e), the Euler characteristic is −4, −8, and −20, respectively. Here, we consider the half of total Euler characteristic *X*/2 as a sum of Euler characteristics of all pores

1 $$X/2=\sum _{n=1}^{N}(1-g)$$

Since the sum of genus $\sum g$ is approximately equal to the total number of holes *G*, Eq. (1) becomes *X*/2 ≈ *N* − *G*. The evolution of interfacial topology is, then, described by using the total Euler characteristic.

Using the Gauss-Bonnet theorem, the total Euler characteristic is calculated from the integral of Gaussian curvature *K* = *κ*_1_ *κ*_2_ over all pore surfaces

2 $$X/2=\frac{1}{4\pi }{\int }_{A}KdA\mathrm{.}$$

**Figure 3:**
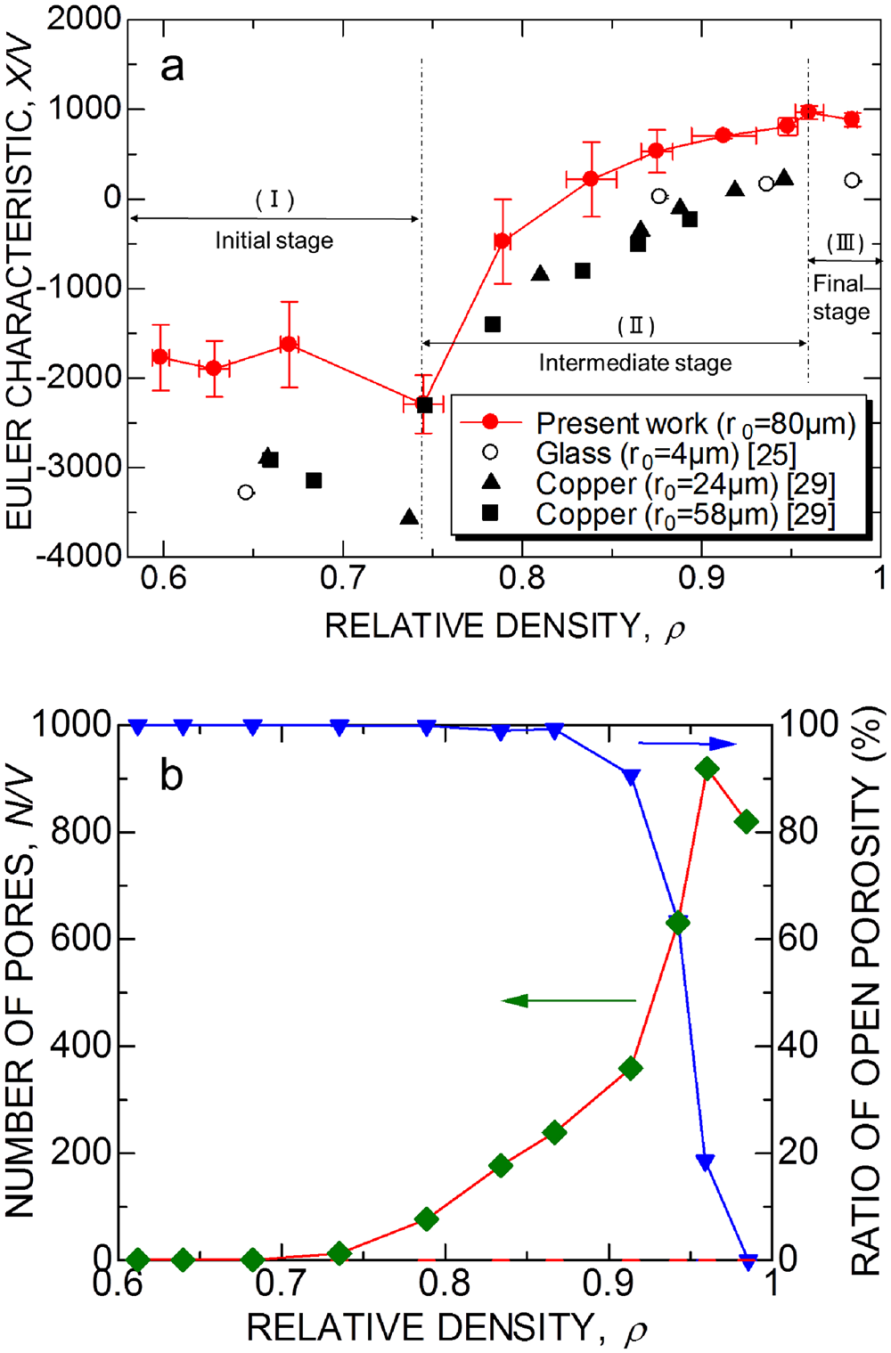
Euler characteristics and number of pores. (**a**) Normalized Euler characteristic per unit volume *X*/*V* and stages of sintering. ((I) Initial stage, (II) intermediate stage, and (III) final stage). (**b**) Number of closed pores per unit volume and the ratio of open porosity. The unit volume is a cube with edge length *L*, which is 10 times of the average particle diameter.

The normalized Euler characteristic per unit volume *V* is given as

3 $$X/2V=\bar{K}{S}_{V}/4\pi $$

where $\bar{K}$ is the average Gaussian curvature (Supplementary Fig. S1), *S*_*V*_ = *A*_*pore*_/*V* is the specific surface area (Fig. 3 in ref. (27)), and *A*_*pore*_ is the total surface area in the unit volume. *X*/2*V* is calculated from the microtomography data, and is plotted as a function of relative density in Fig. 3a. The unit volume *V* is defined as a cube with edge length *L* = 2_0_ *r*_0_, where *r*_0_ is the initial particle radius. Three stages of sintering can be distinguished by using Euler characteristic. In the initial stage of sintering, Euler characteristic is negative, and decreases slightly with relative density up to ρ = 74%. The intermediate stage is the region where Euler characteristic increases with relative density until it has a maximum value (positive) at ρ = 96%. The final stage is characterized as a region where Euler characteristic decreases to zero ultimately. These quantitative criteria for distinguishing stages of sintering agree to the classical qualitative definition based on microstructures fairy well (see Fig. 1).

The changes of Euler characteristic can be analyzed by comparing the number of closed pores *N*/*V*, which is plotted in Fig. 3b. The increase in the number of closed pores shows that they are formed continuously during both the initial and the intermediate stages, although formation rate is limited during the initial stage (<ρ = 74%). This result indicates that the slight decrease of Euler characteristic occurs because the rate of formation of holes is higher than that of hole closure in the initial stage. New holes are created as new contacts with neighbor particles are formed during densification. At the beginning of intermediate stage the increase of Euler characteristic occurs due to hole closures. The rate of hole closure decreases with densification, while the number of closed pores increases. The maximum Euler characteristic at ρ = 96% is almost the same with the number of closed pores. The ratio of open porosity to total porosity (1-ρ) is also plotted in Fig. 3b. The ratio of open porosity decreases significantly as large closed pores are formed. In the final stage of sintering, the Euler characteristic decreases as the number of closed pores decreases.

### Transition between different stages

**Figure 4:**
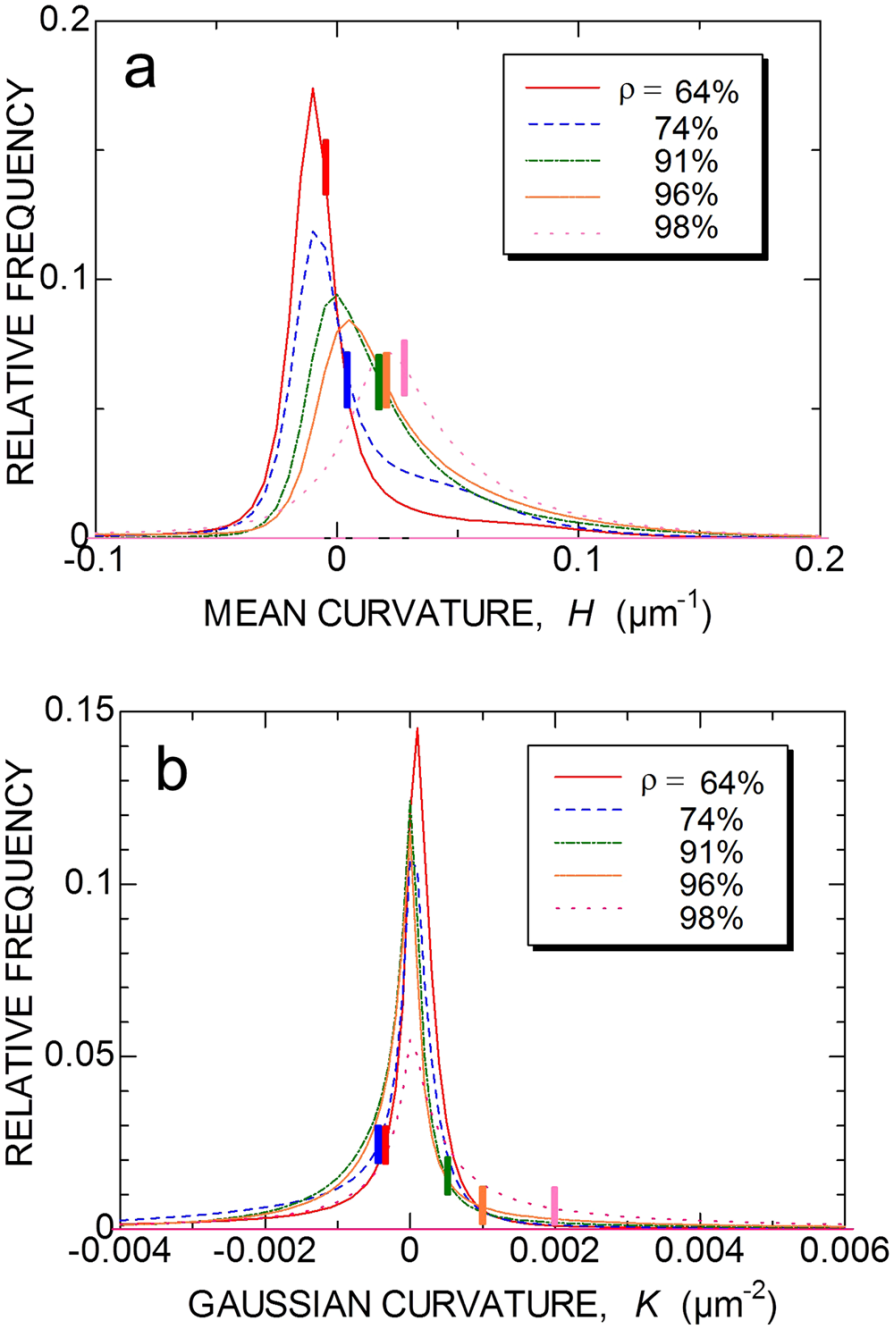
Distribution function of curvatures on pore surface. (**a**) Mean curvature *H* = (*κ*_1_ + *κ*_2_)/2, (**b**) Gaussian curvature *K* = *κ*_1_ *κ*_2_. The mean curvature is defined as positive for a spherical pore and negative for a spherical particle.

The transition from the initial to the intermediate stage is related to an evolution towards a system of interconnected channels with more or less constant curvature in the conventional approach. However, the areal distribution functions of mean curvature and Gaussian curvature (Fig. 4) show that the heterogeneity in curvature does not seem to be a pertinent parameter for distinguishing stages of sintering. Both mean curvature (Fig. 4 in ref. (27)) and Gaussian curvature (Supplementary Fig. S1) increase with relative density monotonously, so that they do not distinguish stages. On the other hand, the transition from the initial to the intermediate stage can be distinguished clearly as an augment of the Euler characteristic.

## Discussion

### Comparison with other sintering mechanisms

Aigeltinger and DeHoff^29^ analyzed the number of holes *G* and the number of pores *N* in sintering of copper particles by using quantitative microscopy. We calculated the Euler characteristic by using their data, and plotted in Fig. 3a for comparison. The Euler characteristic curves are of similar shape, when they are normalized by the average particle size. Three stages of sintering can be distinguished by using Euler characteristic not only for viscous sintering of glass but also for sintering of copper particles by diffusion.

It should be noted that sintering of amorphous and sintering of crystalline materials are rather different. The particle coarsening results from the surface motion in sintering of crystalline materials by evaporation-condensation and surface diffusion. The grain growth results from grain boundary motion by curvature. Although both coarsening and grain growth affects microstructure evolution in sintering of crystalline materials^7^, such effects are not involved in viscous sintering. Nevertheless, as far as we focus on topological nature of microstructure, common features are observed for sintering of both amorphous and crystalline materials.

The effect of particle size on viscous sintering of glass particles can be predicted by using scaling law of Herring^34^, thereby, we suppose the microstructure is self-similar for different particle size. In Fig. 3a, the normalized *X*/2*V* in viscous sintering of large glass particles (*r*_0_ = 80 μm, present work) was compared with that in sintering of small glass particles (*r*_0_ = 4 μm), which was observed by synchrotron X-ray microtomography^25^. Euler characteristic vs relative density curves were self-similar despite the difference in particle size.

The sintering of crystalline materials is affected significantly with the decrease of particle size into the nanometer range^35^. The particle rotation and sliding contribute to densification in the initial stage of sintering of loosely packed nanocrystalline powder^36^. Common features observed in sintering of coarse particles may not be observed in sintering of such loosely packed powder. For example, Schleef and co-workers^37^ reported that Euler characteristic increased monotonically in sintering of fresh snow with relative density of 0.1. However, at present, the voxel resolution (0.28–2.5 μm) of X-ray microtomography is insufficient to study sintering of nanocrystalline particles.

### Euler characteristic per unit volume

Consider the initial stage of sintering of identical spheres periodically arranged in a simple cubic lattice. Because there is one unique interconnected pore (*N* = 1 in Eq. (1)), the half of total Euler characteristic is a sum of genus made on the porous cells $X/2=1-\sum g$. The periodic cubic cell structure is topologically the same with Schwartz P surface, which has the genus 3 for a unit cell (Supplementary Fig. S3a). The average number of particles *P* in the unit volume is given as *P* = 6000*ρ*/*π* where *ρ* is the relative density. At *ρ* = 0.6, the normalized Euler characteristic per unit volume *X*/2*V* is about −2800, which is in good agreement with experimental results in Fig. 3a. The Euler characteristic per unit volume depends on the particle shape, the distribution function of particle size, and that of pore size. It is clearly shown that the number of small pores, where the pore size is normalized by the average particle radius, is larger in sintering of large particles than in the sintering of small particles (see Supplementary Fig. S2). This is partly due to the difference in relative resolution (the ratio of voxel size to the particle radius), which is 0.03 and 0.07 for the sintering of large particles (present work) and the sintering of small particles, respectively. It should be noted that Euler characteristic is sensitive to the relative resolution, because small bodies are weighted equally with large bodies.

#### Simulation of topological evolution

**Figure 5:**
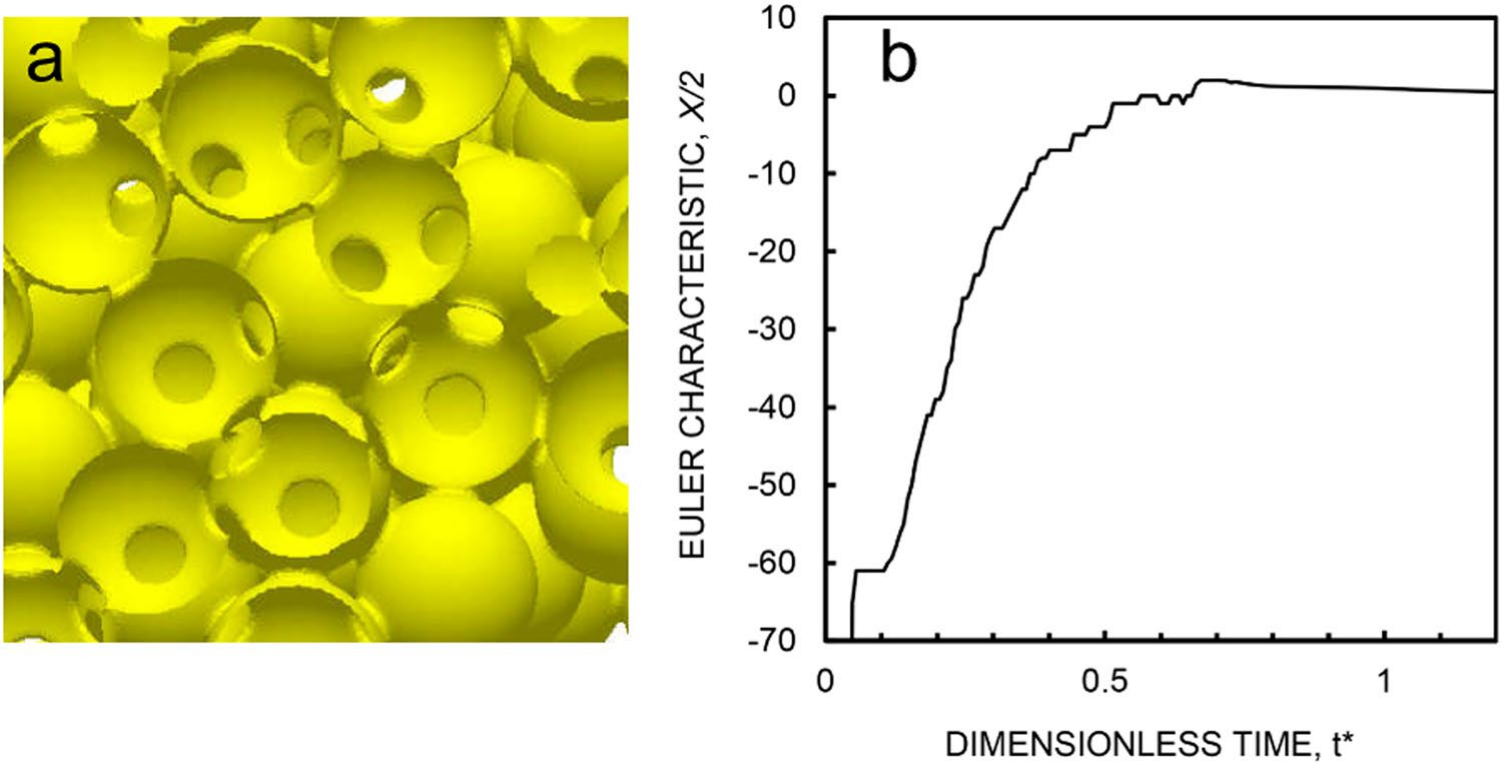
Computer simulation of sintering by bulk diffusion. (**a**) Snap shot at the initial stage, (**b**) Euler characteristic.

In order to visualize the topological evolution in sintering, we performed a computer simulation using a mathematically simple model for the case the grain boundary energy is zero, so as to compare with the microstructure evolution in viscous sintering. The computer simulation of sintering was conducted by assuming a case where the bulk diffusion is so fast that the sintering rate is controlled by the rate of creation/annihilation of vacancies on the surface^38, 39^ (See Supplementary Methods). Closed pores shrink by bulk diffusion from surface to pore surface^6^. Figure 5a and Supplementary Movie 3 show the evolution of pore structures in sintering of a cluster of 128 spheres. The movie clearly shows that large closed pores are formed by consecutive pinch-off of pore channels in a similar way to the microstructural evolution in viscous sintering. Supplementary Figures S4 illustrate how a void and pore channels evolve from the topological cell of particles. Figure 5b shows the Euler characteristic varies with a dimensionless time. The Euler characteristic increased after a plateau region, reached to a maximum, and decreased toward zero. This topological feature agreed with experimental observations qualitatively, although sintering mechanisms differed with each other.

#### Topology and sintering mechanics

The sintering mechanics depends on the topology of microstructure. For the initial stage, large scale simulations of sintering of many particles have been successfully achieved by using discrete element method (DEM) recently^40–42^. The mechanics underlying this method is a relationship between the relative velocity of particles and the sintering force acting among neighbor particles in both sintering by grain boundary diffusion^20, 43^ and in viscous sintering^44^. The macroscopic sintering stress can be estimated from the microscopic sintering force, which is a function of the average contact radius and the average particle coordination number^27^. In the final stage where closed pores are dispersed randomly, each pore has a local sintering stress^38^. For viscous sintering, the macroscopic sintering stress is defined as a volume average of local sintering stress of pores^45^. It is simply calculated from the relative density and the specific surface area^25^. The present authors have shown that sintering stress can be derived as functions of relative density directly from the knowledge of microstructure observed by X-ray microtomography^25, 27^, and proposed a method to calculate the sintering stress in the intermediate state by using the average curvature of pore surface. The range in application of these three methods can be clearly defined by distinguishing stages of sintering from Euler characteristic.

We conclude that three stages of sintering are distinguished by using Euler characteristic, which is given as the number of closed pores minus the genus. The random packing of particles is expressed as vertices, edges, faces, and cells topologically. The genus is the number of holes, which is equivalent to the number of pore channels connecting voids inside each cell. The elementary processes in morphological transformation of pore structure is the creation and annihilation of pore channels and those of closed pores. Although the Euler characteristic vs relative density curve was studied only for viscous sintering of silicate glass particles here, we believe the result is general and may explain the sintering behavior of many ceramic and metallic particles.

These results may open the way to control internal defects formed during sintering, because it is crucial to understand the evolution of heterogeneous pore structures for improving the mechanical reliability of products. The interfacial topology provides a description of stages of sintering, and helps to recognize the roles of forces behind the microstructural evolution.

## Methods

### Materials

Sintering of glass particles and X-ray microtomography have been fully described in ref. 27 and will be outlined here. The soda lime glass powder used in this work consisted of spherical particles (Spheriglass GB-AD, Potters Industries). It had a composition of 72.0 wt% SiO_2_, 13.5 wt% Na_2_O/K_2_O, 9.0 wt% CaO, 3.4 wt% MgO, 2.0 wt% Al_2_O_3_, and 0.1 wt% Fe_2_O_3_. The glass particles were sieved to obtain a homogeneous particle size distribution between 155 and 183 μm in diameter. We assumed the average radius of 80 μm for the polydispersed particles. This powder was mixed with polyvinylalcohol (PVA) and surfynol, and the resulted aqueous slurry (60 vol% solid content) was casted on an alumina substrate using a doctor blade. The dried green sheets were removed from the substrate and cut to the desired sample size (4.5 × 6.8 × 3.0 mm). Binder burnout and calcination were conducted by heating the sample at a rate of 3 °C/min up to 450 °C, and 5 °C/min up to 690 °C, then held for 30 minutes. The isothermal sintering treatment was performed in an external furnace, and then, taken off for microtomography measurement. This step was repeated for one sample. In each step, the sample was heated at 5 °C/min and held at 690 °C in air for 30 minutes.

### Tomography

The sample was analyzed by X-ray computed microtomography (Bruker, SKYSCAN 1172). The X-ray source was set at a voltage of 80 kV and a current of 100 μA. The sample was rotated by steps of 0.1° up to 180°. The 3-D mappings with voxel size 2.5 × 2.5 × 2.5 μm were reconstructed from the acquired data by using the filtered back-projection method. The 3-D visualization and geometrical measurements were performed using Amira (VSG), and a Gaussian filtering was applied to reduce the noise in 2-D images. Local thresholding method was used to segment the gray value image into pore and material, so as to determine the pore volume. The pore surface was discretized using triangular meshing, from which the pore area was calculated. Curvature on each triangle was calculated from the eigen-values and eigen-vectors of the quadratic form.

## Electronic supplementary material

Supplementary Movie 1

Supplementary Movie 1c

Supplementary Movie 2a

Supplementary Movie 2b

Supplementary Movie 3

Interface topology for distinguishing stages of sintering
