# Supplementary material for: Interface topology for distinguishing stages of sintering
Source: Sci Rep. 2017 Sep 11;7:11106. doi: 10.1038/s41598-017-11667-2 (PMC5593873; doi:10.1038/s41598-017-11667-2)
Supplement: Supplementary file 6 — Interface topology for distinguishing stages of sintering [file 41598_2017_11667_MOESM6_ESM.doc]

**Interface topology for distinguishing stages of sintering**

Gaku Okuma^1^, Daiki Kadowaki^1^, Tsuyoshi Hondo^2^, Satoshi Tanaka^2^, and Fumihiro Wakai^1^

^1^ Laboratory for Materials and Structures, Institute of Innovative Research, Tokyo Institute of Technology, R3-23 4259 Nagatsuta, Midori, Yokohama, 226-8503, Japan

^2^ Department of Materials Science and Technology, Nagaoka University of Technology, 1603-1, Kamitomioka, Nagaoka, Niigata 940-2188, Japan

**Supplementary Information**


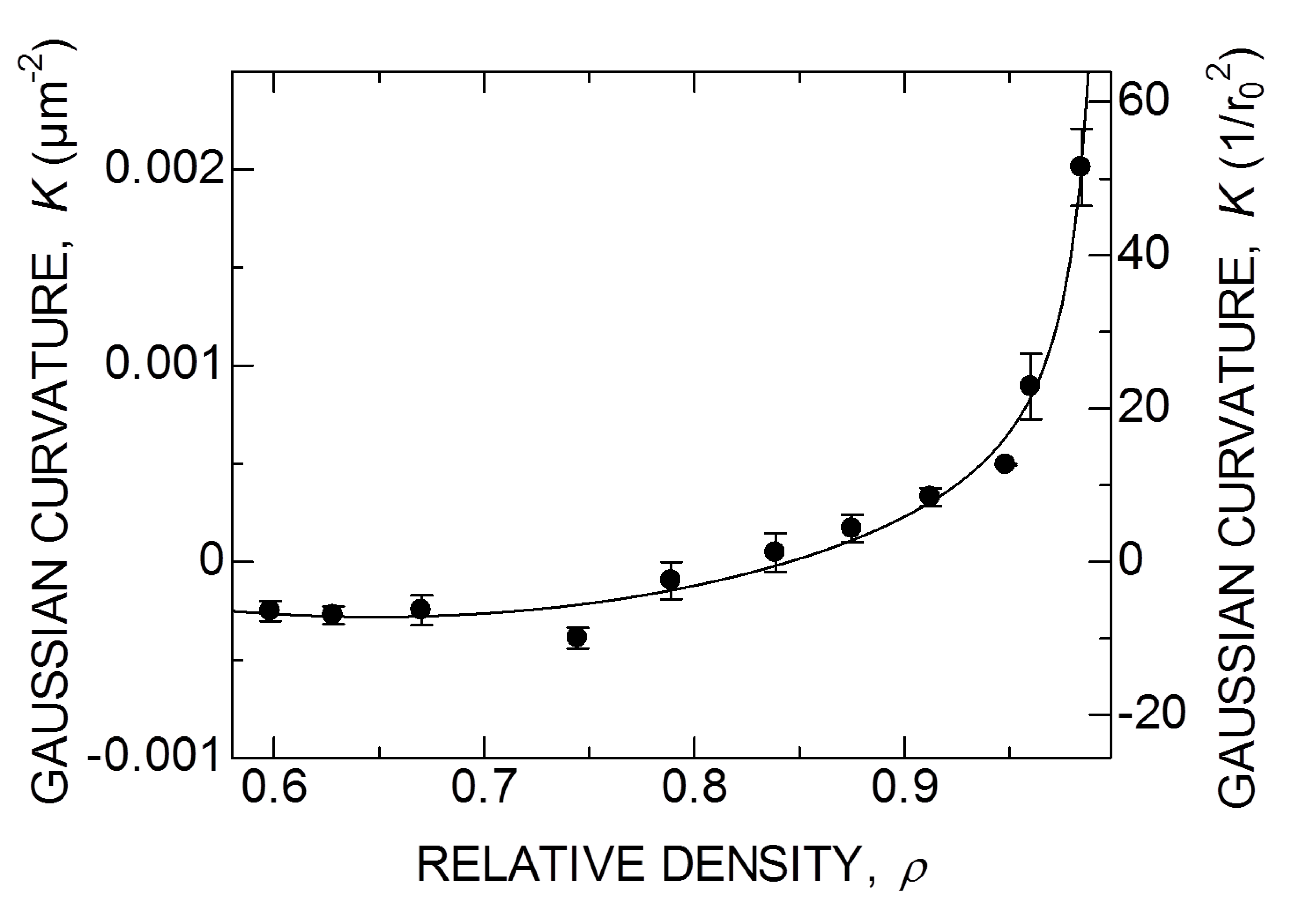


**Supplementary Figure S1** | **Average Gaussian curvature $K$ on pore surface as a function of relative density.** The local curvature on pore surface varies widely, so that we choose large volume elements with 1.5 mm to define the average. The error bar indicates the heterogeneity inside the specimen (4.5 mm × 6.8 mm × 3.0 mm).


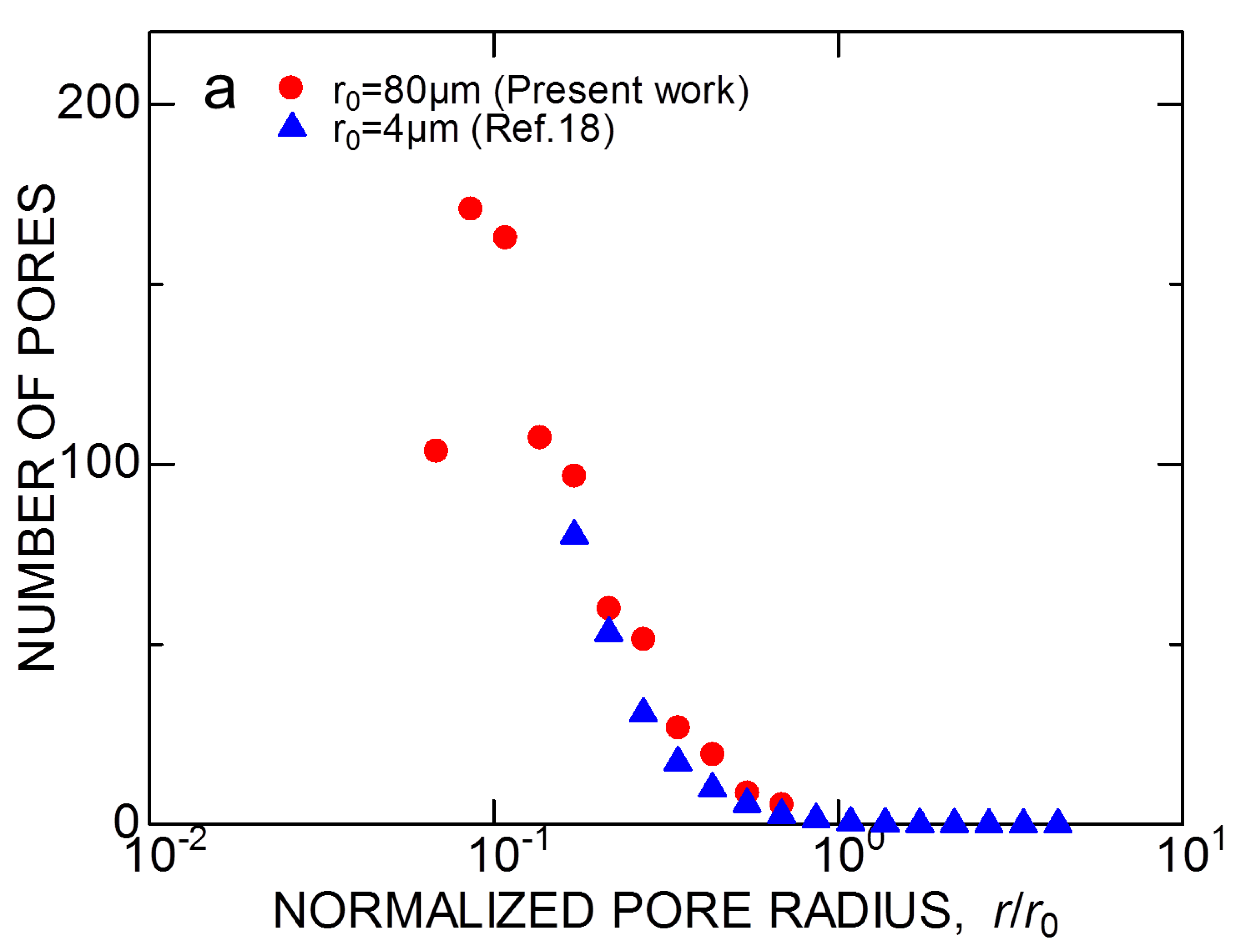


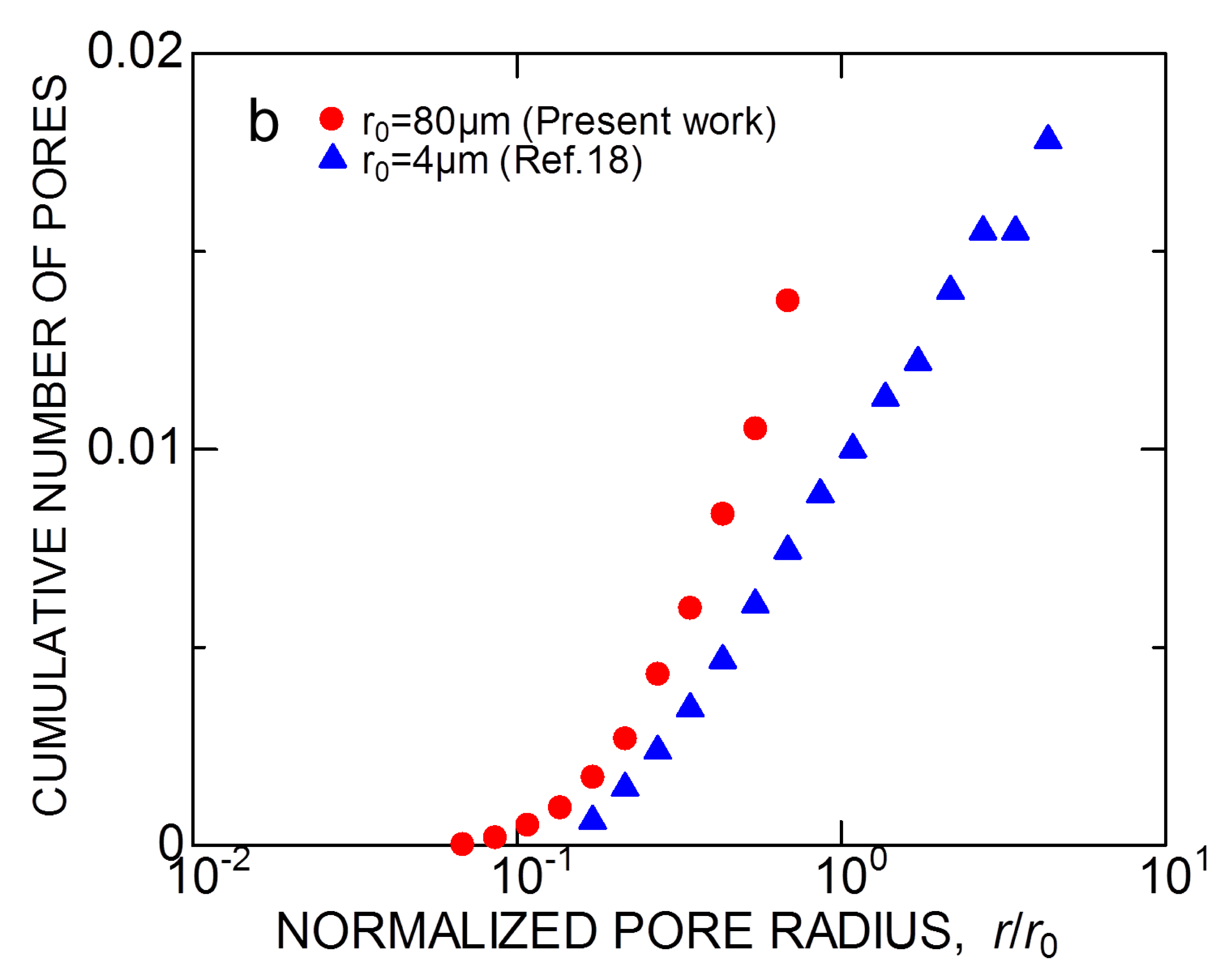


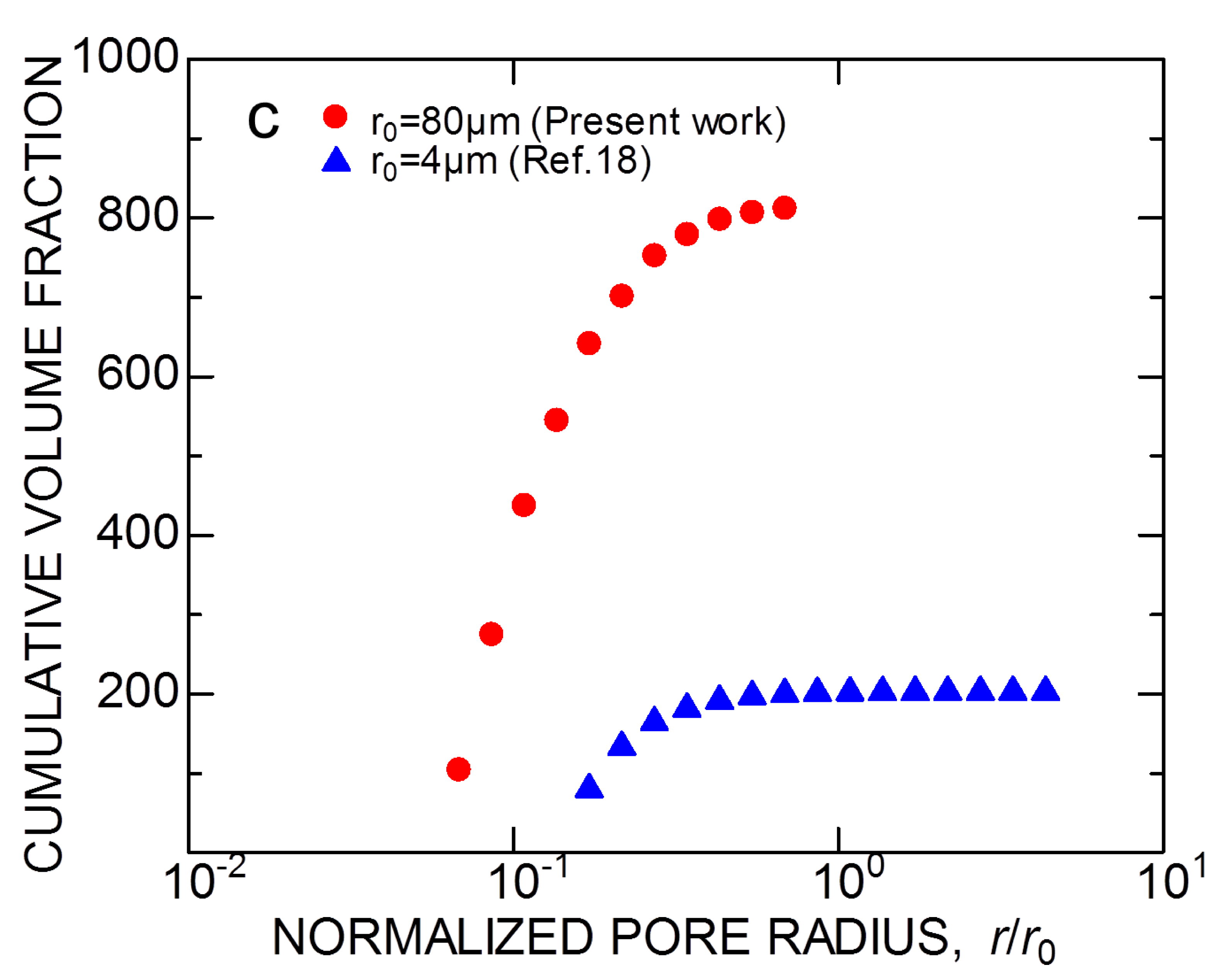


**Supplementary Figure S2** | **Normalized pore size distribution in the final stage of sintering.** (a) The number of pores in the unit volume, (b) Cumulative number of pores, (c) Cumulative pore volume fraction.

**a b**

**
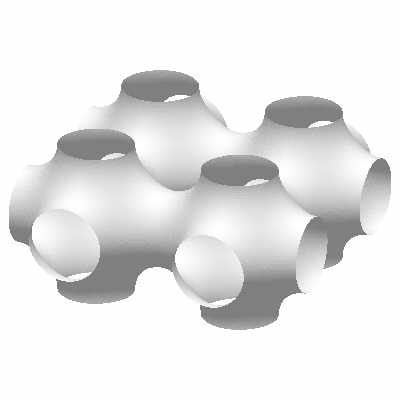
**

**
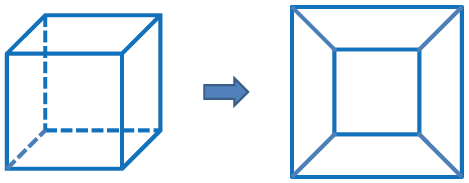
**

**Supplementary Figure S3** | **Genus of cubic structures.** (a) Schwartz P surface with genus 3, (b) A cubic cluster of particles. For an isolated cluster (left model), we can stretch the top hole so that it is large enough. Then, collapse the top portion to flatten the model (right model). The number of through holes is actually five, and then the genus is 5 ($\chi=-8$ in Fig. 2 d).

**
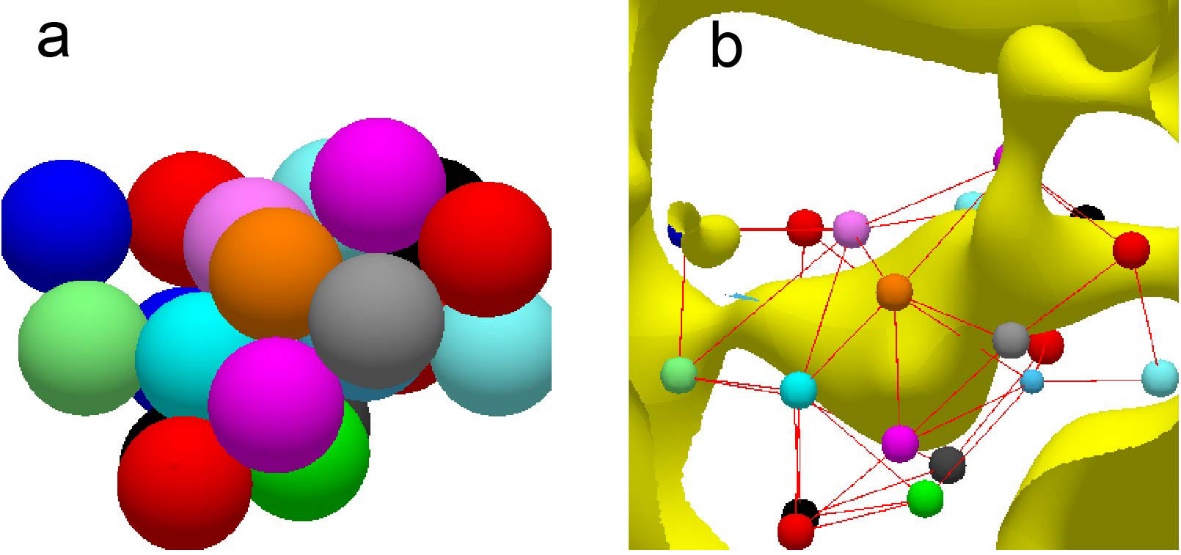
**

**Supplementary Figure S4** | **Computer simulation of sintering by bulk diffusion.** (a) An example of initial particle packing, (b) A void and pore channels together with the topological cell model.

**Supplementary Methods**

**Computer simulation of sintering by interface-controlled bulk diffusion**

As a mathematically simple sintering mechanism, we consider a model, where the bulk diffusion is so fast that the shrinkage rate is controlled by the interface reaction (creation and elimination of vacancies by the surface)^38^. We assume grain boundary is not the source/sink of vacancy for simplicity. Closed pores can shrink by bulk diffusion from surface to pore surface. The chemical potential in the bulk will be uniform, if the bulk diffusion is infinitely fast. It is supposed that the interface reaction rate is proportional to the difference in chemical potential between the bulk and the surface. The surface moves inward when a vacancy is created on the pore surface. The normal velocity of surface is expressed as

(S1)

where $M$ is the mobility, $\gamma_{s}$ is surface energy, $\kappa=2H$ is curvature, and $\kappa_{\text{av}}$ is the average curvature. The detailed derivation of Eq. (S1) is given in Appendix D of Ref. 38.

Brakke’s Surface Evolver program^46^ was used to simulate the microstructural evolution in sintering by interface-reaction controlled bulk diffusion. The outline of the program is described here briefly. Both the surface and the grain boundary of particles are represented as a set of triangular finite elements, or facets. Each facet consists of three edges and three vertices. The surface and the grain boundary have energies proportional to their area. The Surface Evolver program evolves the surface toward minimal energy by a gradient descent method under any constraint. The gradient of energy at a vertex is a force, which must be converted to a velocity vector for the motion. This conversion involves what may be called the mobility factor: how a vertex responds to the force on it. In the interface-reaction controlled sintering, the resistance of motion is actually due to the surface, not the vertex. In order to approximate this, the resistance to motion of a vertex is proportional to the area associated with vertex. The actual motion is found by multiplying the velocity by a scale factor. The physical interpretation of the scale factor is the time step. The surface motion can be approximated by enforcing the constraint on conservation of the total volume of particles. The Surface Evolver program had been applied to simulate the interface-reaction controlled sintering^38,39^, for example, evaporation-condensation, interface-reaction controlled bulk diffusion, and interface-reaction controlled surface diffusion.

We simulated the sintering of a cluster of 128 spherical particles. The result was analyzed by using the dimensionless time defined as , where $r_{0}$ is the initial radius.

**Supplementary References**

46. Brakke K. A. The Surface Evolver. *Exp. Math.* **1**, 141-165 (1992).
